# Supplementary material for: Selective filtering of multi-photon events from a single-photon emitter
Source: Nat Commun. 2026 Aug 14;17:8412. doi: 10.1038/s41467-026-76358-x (PMC13476227; doi:10.1038/s41467-026-76358-x)
Supplement: Supplementary file 1 [file 41467_2026_76358_MOESM1_ESM.pdf]

Supplementary Information: Selective filtering of multi-photon events from a single-photon emitter

Friedrich Sbresny,[1,2,3,*] Carolin Calcagno,[1,2,3] Sang Kyu Kim,[1,2,3] Katarina Boos,[1,2,3] William Rauhaus,[1,2,3] Frederik Bopp,[2,4,3] Hubert Riedl,[2,4,3] Jonathan J. Finley,[2,4,3] Eduardo Zubizarreta Casalengua,[1,2,3] and Kai Müller[1,2,3]

[1] *TUM School of Computation, Information and Technology, Technische Universität München, 85748 Garching, Germany*
[2] *Walter Schottky Institut, Technische Universität München, 85748 Garching, Germany*
[3] *Munich Center for Quantum Science and Technology (MCQST), Schellingstr. 4, 80799 München, Germany*
[4] *TUM School of Natural Sciences, Technische Universität München, 85748 Garching, Germany*

SUPPLEMENTARY NOTE 1: DETAILS OF THE THEORETICAL MODEL

A. Time-integrated photon correlations

We are particularly interested in the time-integrated two-photon correlations

$$g^{(2)}[0;\Gamma] = \frac{\int_0^T \int_0^T \mathcal{G}_\Gamma^{(2)}(t_1,t_2)\, dt_1 dt_2}{(\int_0^T n_\Gamma(t)\, dt)^2}\,, \tag{1}$$

where T, the integration time, is taken to infinity ($T \to \infty$). The frequency-filtered population and the second-order Glauber correlation function are defined in terms of the sensor correlations (see Methods) as

$$n_\Gamma(t) = \left(\frac{\Gamma}{2\epsilon}\right)^2 \langle \varsigma^\dagger(t)\varsigma(t)\rangle\,, \tag{2}$$

and

$$\mathcal{G}_\Gamma^{(2)}(t_1,t_2) = \left(\frac{\Gamma}{2\epsilon}\right)^4 \langle \mathcal{T}_-[\varsigma^\dagger(t_1)\varsigma^\dagger(t_2)]\mathcal{T}_+[\varsigma(t_2)\varsigma(t_1)]\rangle\,, \tag{3}$$

where $\mathcal{T}_\pm$ chronologically orders the operators from right to left, and left to right, respectively. The numerator and denominator of Eq. (1), that is, the time-integrated two-photon correlation and population, respectively, can be efficiently computed using the methods developed in Ref. [1]. When the filter is large enough, that is, $\Gamma \to \infty$, we recover the unfiltered photon statistics. In particular, for the two-level system emission, the correlation function converges to

$$\mathcal{G}_\sigma^{(2)}(t_1,t_2) = \mathcal{G}_{\Gamma\to\infty}^{(2)}(t_1,t_2) = \langle \mathcal{T}_-[\sigma^\dagger(t_1)\sigma^\dagger(t_2)]\mathcal{T}_+[\sigma(t_2)\sigma(t_1)]\rangle\,, \tag{4}$$

which corresponds to the result shown in Fig. 1a.

B. Biexciton ladder polarization

As explained in the Methods section, the biexciton ladder is coupled to the two linear polarizations, that is, $X = \sigma_{21} + \sigma_{42}$ couples to the H polarization, whereas $Y = \sigma_{31} + \sigma_{43}$ couples to the V polarization. The input laser field can have any arbitrary polarization, characterized by the vector $\hat{\boldsymbol{u}}_{\rm in}$

$$\hat{\boldsymbol{u}}_{\rm in} = \begin{pmatrix} \cos\theta \\ \sin\theta\, e^{i\phi} \end{pmatrix}\,, \tag{5}$$

* Corresponding author: friedrich.sbresny@tum.de

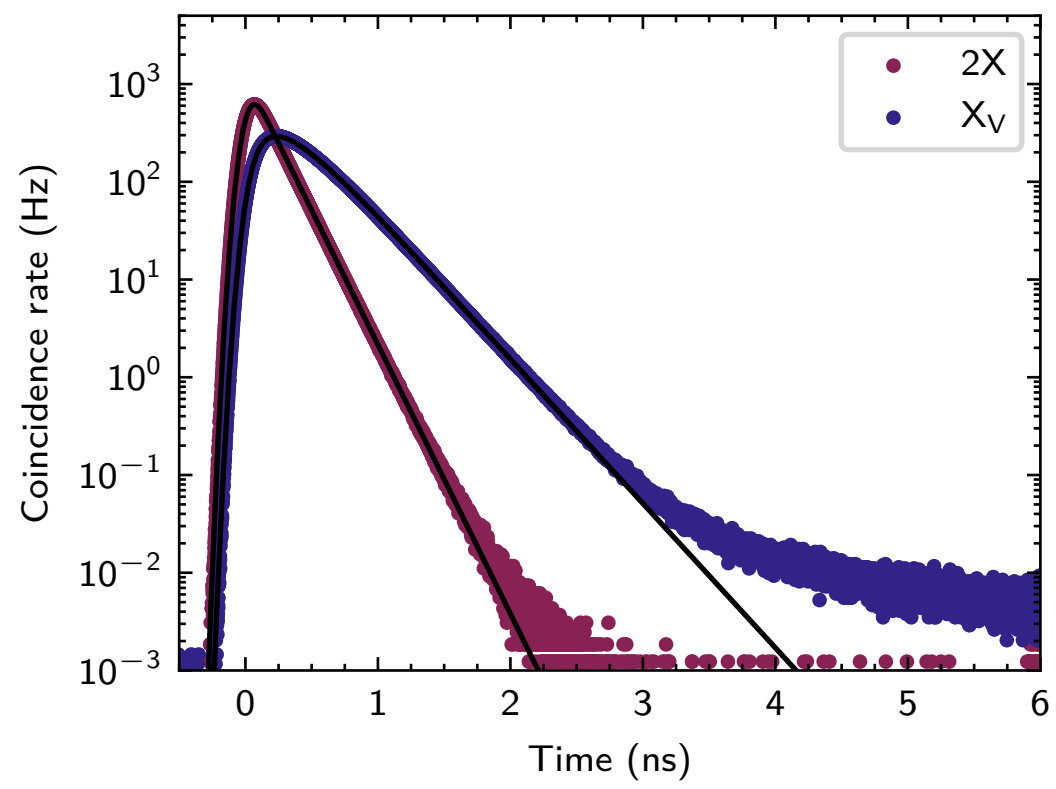

Supplementary Figure 1: Lifetime measurements. Time-resolved biexciton (wine) and exciton (indigo) photoluminescence upon excitation of the biexciton with a π-pulse. The exciton state population slowly builds up as the biexciton decays. Fitting the two curves (black lines), we extract $\tau_{2X} = 158\,\text{ps}$ and $\tau_{X_V} = 294\,\text{ps}$.

which includes all the polarization states we will consider (fully polarized light), such as linearly polarization $\hat{\boldsymbol{u}}_\theta$ (with $0 \leq \theta < \pi$ $\phi = 0$) and circularly (left and right) polarized light $\hat{\boldsymbol{u}}_{L,R}$ (with $\theta = \pi/4$ and $\phi = \pm\pi/2$, respectively). As we previously discussed, each branch of the exciton ladder couples to the horizontal and vertical components of the light field, then the effective amplitude of each branch is $\Omega_{H,V}(t) = \Omega_0(t)(\hat{\boldsymbol{u}}_{in}^* \cdot \hat{\boldsymbol{v}}_{H,V})$, where $\hat{\boldsymbol{v}}_H = \hat{\boldsymbol{u}}_0$ and $\hat{\boldsymbol{v}}_V = \hat{\boldsymbol{u}}_{\pi/2}$, which is obtained by projecting the input polarization vector onto the horizontal and vertical vectors, and $\Omega_0(t)$ is the effective laser amplitude. For all the computation, we have assumed the laser polarization to be horizontal.

SUPPLEMENTARY NOTE 2: LIFETIME

We measure the lifetimes of the two transitions of the biexciton-exciton cascaded decay upon excitation of the biexciton via the two-photon transition with a pulse of area π and length 5 ps. A photodiode signal derived from the laser pulse is correlated with either the biexciton (wine dots) or exciton (indigo dots) emission as shown in Supplementary Figure 1. To filter the emission, we use a spectral filter with a width $\Gamma_\nu = 98\,\text{GHz}$. We fit the curves with a convolution of the solution to their coupled rate equations and a Gaussian function (black lines). Here, the width of the Gaussian which corresponds to the timing resolution of our setup is left as a free fit parameter. For the biexciton emission this yields the expected monoexponential decay with a lifetime $\tau_{2X} = 158\,\text{ps}$. For the exciton we observe the expected buildup and subsequent decay and extract a lifetime of $\tau_{X_V} = 294\,\text{ps}$. In addition, the measurement exhibits a very weak second decay, which which we attribute to a very low but finite probability of a spin flip and thus creation of a dark exciton [2].

SUPPLEMENTARY NOTE 3: SIGNAL-TO-NOISE RATIO

To be able to resolve the instantaneously emitted photon in the second-order correlations, it is crucial to maximize the signal-to-noise ratio of the experiment. To optimize this ratio, we chose a specific quantum dot transition (the exciton emission in a cascaded four-level system) and apply multiple polarization and spectral filtering techniques, as described in the manuscript. To ensure that we are measuring the instantaneous photon instead of undesired correlation events from signal and noise, we investigated the origin of the residual noise in our setup and identified it to be dominated by laser leakage into the emission signal. Therefore, right before each correlation measurement, we took noise count rate measurements with the quantum dot turned out of the photoluminescence regime by biasing the sample diode, but with the excitation pulse turned on. The measured count rate includes both the residual laser leakage counts and the intrinsic dark count rate of the detectors, which is in our case, due to the use of low noise superconducting nanowire detectors, lower than the laser leakage count rate. For the measurements in the manuscript, the typical ratios between noise counts and quantum dot counts lie between 1:20000 and 1:400000. Only for the measurement using the widest filter and the shorter pulse length, significant amounts of laser light start to leak in, resulting in deterioration of the ratio to 1:3400. To estimate the minimum obtainable $g^{(2)}[0;\Gamma_\nu]$, we performed a Monte-Carlo simulation, where we assumed a perfect single-photon source without any two-photon events. We use the measured quantum dot count rates and an additional Poissonian noise source with the measured laser leakage count

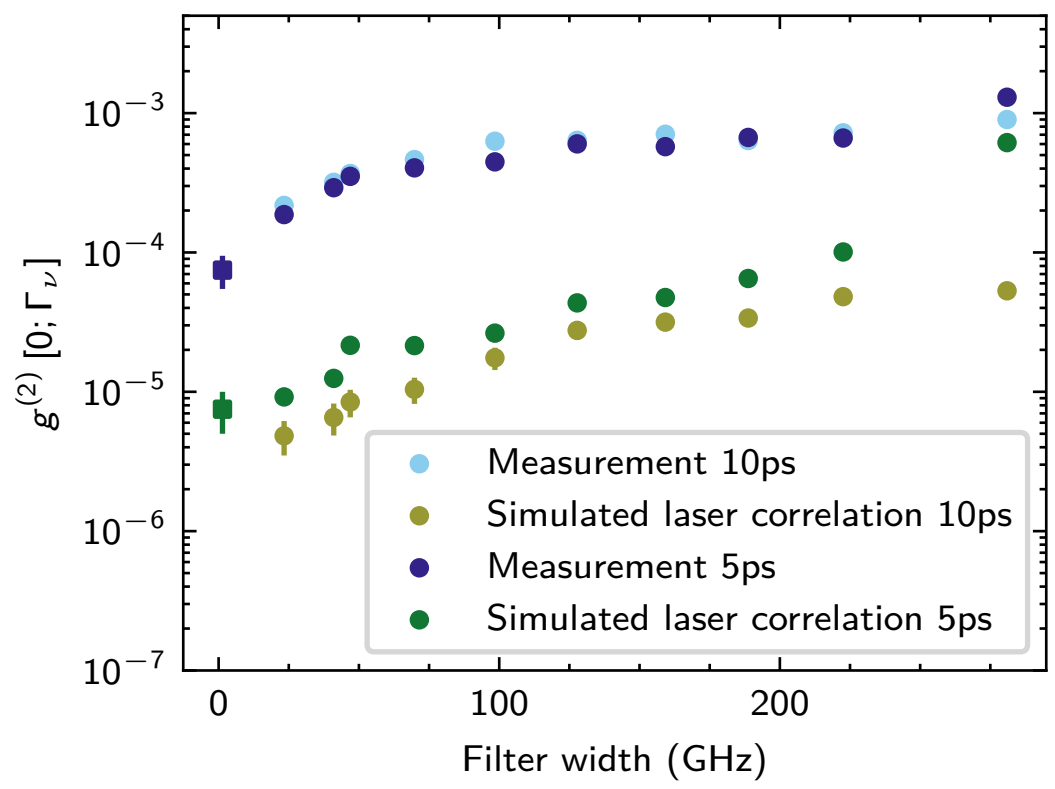

Supplementary Figure 2: Signal-to-noise ratio estimation. Measured data of the filter dependent $g^{(2)}[0;\Gamma_\nu]$ from Fig. 3a of the main manuscript (indigo and cyan). The green and olive data points represent our lower bound estimate of the $g^{(2)}[0;\Gamma_\nu]$, which is derived from the simulated correlations of the laser leakage and a perfect single-photon source. The error bars represent the 1σ confidence interval determined from Poissonian counting statistics.

rate. We determine the resulting $g^{(2)}[0;\Gamma_\nu]$ by simulating the autocorrelation of the photon stream in a Hanbury Brown Twiss experiment, which is displayed by the green and olive data points for pulse durations of 5 ps and 10 ps in Supplementary Figure 2, respectively. For all data points but one, we observe that our estimate of the lowest possible $g^{(2)}[0;\Gamma_\nu]$ with the measured noise of our experimental setup is significantly lower than the experimentally measured values. This means that the second-order coherence measurements presented in the manuscript are dominated by the intrinsic multi-photon error rate of the system and not by coincidences with dark or laser leakage counts. Only for the short pulse length and the widest filter we observe that the lower bound estimate is of a similar order as the measured $g^{(2)}[0;\Gamma_\nu]$, which negatively affects the measured value.

SUPPLEMENTARY NOTE 4: TRANSMISSION FOR DIFFERENT FILTER FUNCTIONS

As mentioned briefly in the manuscript, the exact filter transmission function strongly affects the transmission of both the wanted single-photon emission and the unwanted instantaneous photon. To discuss this, we filter a Lorentzian signal, representing the single-photon emission, by three different filter functions: a Lorentzian as in the presented simulations, a Gaussian and a Square. Note that the experimentally used filters are across a continuous spectrum of those three filter functions. We define the filter width Γ as the full width at half maximum (FWHM) for the Lorentzian and Gaussian function, and as the full width of the Square function. In the sketch in Supplementary Figure 3a, the black line represents the Lorentzian signal, while the dashed lines represent the three filter functions. The integrated transmission of the signal multiplied with the filter function is plotted against the semilogarithmic filter width in Supplementary Figure 3a. For filter widths larger than the radiative emission linewidth γ, the Square filter function allows for the highest total transmission, while for filter widths below the radiative linewidth γ, the Lorentzian filter allows for the highest transmission. Note that for all filter shapes, the transmission of the signal is at approximately 50% at one time the filter width, while at 10 times the filter width it rises above 90%. Thus for filters that are significantly larger than the radiative linewidth, the photon loss of the wanted single-photon emission induced by the filter is minimal. This contrasts with the unwanted instantaneous photon: We model this, by filtering a Gaussian emission signal of FWHM γ. Note that this assumption is based on the fact that the first photon needs to be emitted during the duration of the Gaussian laser pulse, and represents only an approximation. The result can be seen in Supplementary Figure 3b, where we are interested in the minimal transmission of the signal. For filter widths larger than γ, the transmission is minimal for the Lorentzian, while for filter widths narrower than γ it is minimal for the Square and Gaussian filters. If the driving pulse duration is chosen to be much shorter than the emission lifetime, this leads to the interesting regime studied in this work: The Lorentzian single-photon emission is filtered at widths much larger than its radiative linewidth, allowing for high transmission of the signal. In contrast the instantaneous photon is filtered with filters much narrower than its FWHM, filtering out a significant part of the undesired emission. Comparing the wide filter regime of Supplementary Figure 3a and the narrow filter regime of Supplementary Figure 3b, one observes that the largest improvement of the $g^{(2)}[0;\Gamma]$ in this regime could be achieved using Square filters, while the smallest improvement can be achieved with Lorentzian filters.

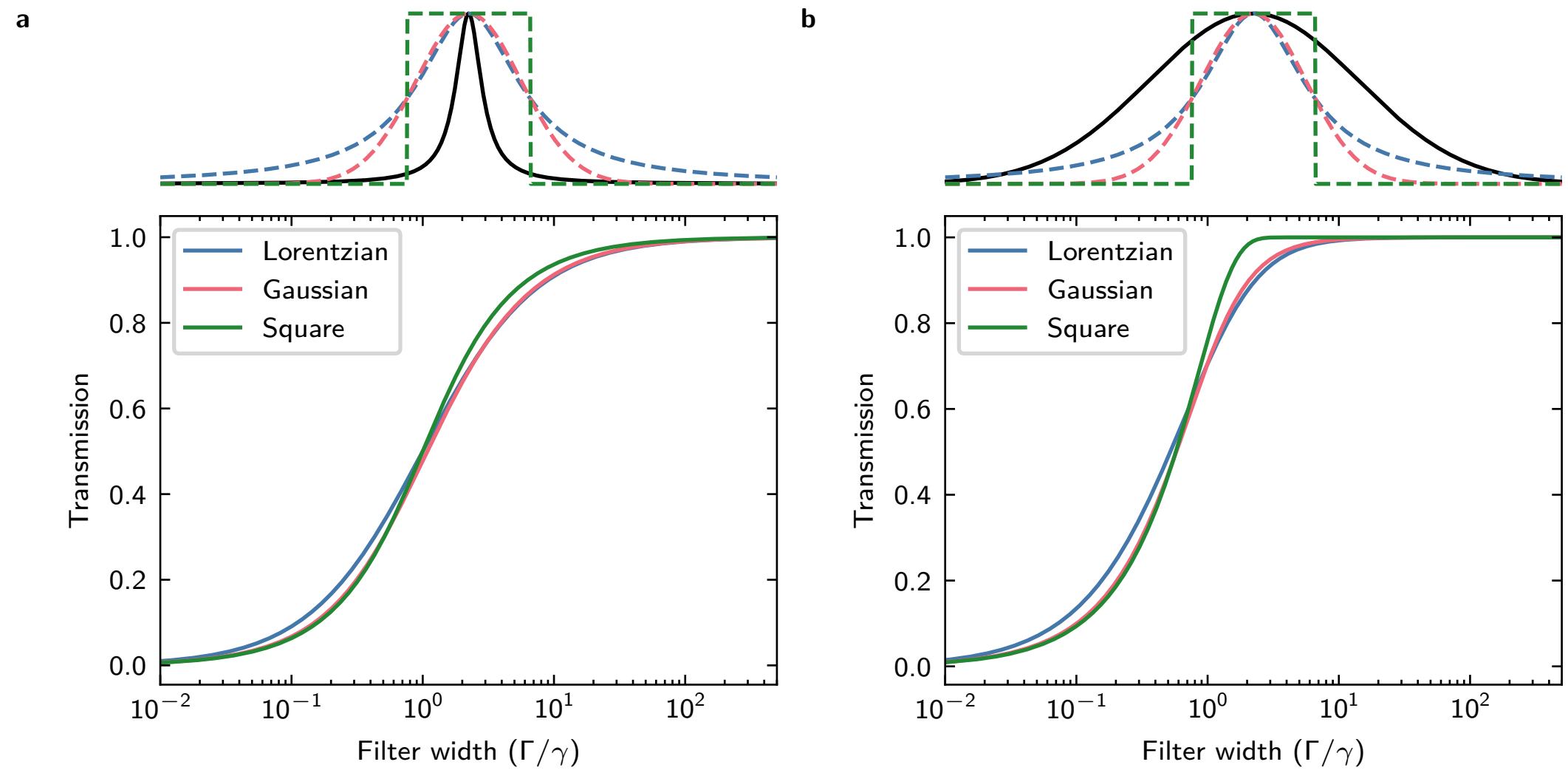

Supplementary Figure 3: Filter function transmission. **a**, Lorentzian signal of FWHM γ representing the single-photon signal (black line) filtered by Lorentzian (blue dashes), Gaussian (red dashes) and Square (green dashes) filters of width Γ. In the plot, the integrated transmission of the signal multiplied with the filter function is displayed against the filter width. For filters wider than the Lorentzian signal's FWHM γ, the Square filter allows for the highest transmission, while for filters narrower than γ, the Lorentzian filter allows for the highest transmission. **b**, Gaussian signal of FWHM γ representing the instantaneous photon filtered by the same filter functions. Here we are interested in the minimal transmission, which is lowest for the Lorentzian filter for widths wider than the instantaneous photon's FWHM γ and for the Square filter for widths narrower than the FWHM.

The optimum filter width for a specific experiment represents a tradeoff between the improvement of the $g^{(2)}[0;\Gamma]$ and the single-photon flux. Simultaneously, the driving pulse duration provides a turning knob to improve the characteristics of this tradeoff: By making the pulse shorter in time, the instantaneous photon spectrum becomes broader, allowing for the filtering of a larger portion of the instantaneous photon without significantly reducing the single-photon emission.

SUPPLEMENTARY NOTE 5: FILTERED $g^{(2)}[0;\Gamma]$ FOR CARVED PULSES

In the manuscript, we identified the non-perfectly Gaussian-shaped laser pulses in the experiment to deteriorate the degree of second-order coherence. The pulses used to excite the quantum dot are derived from a femtosecond laser pulse and two 4f pulse shapers [3] in a serial configuration. In the Fourier plane of the pulse shapers an adjustable mechanical slit is located, whose rectangular aperture of adjustable width is defining the spectral components transmitted and consequently the pulse length. In the time domain, these spectral components yield a sinc function as pulse shape. Note here, that due to the finite size of the focused laser spot in the Fourier plane of the pulse shaper, the edges of the spectral rectangle are smoothed. This results in practice in a close to Gaussian pulse shape, which exhibits broader tails in autocorrelation measurements.

To investigate the impact of this small deviation from a perfect Gaussian pulse shape on the degree of second-order coherence in the simulations, we model the driving pulse field as

$$\Omega_X(t) = \frac{\Theta}{\pi\tau_c \tanh\frac{1}{2\alpha\tau_c}} \frac{\pi\alpha t}{\sinh \pi\alpha t} \frac{\sin\frac{t}{\tau_c}}{\frac{t}{\tau_c}}, \tag{6}$$

where Θ is the pulse area. The parameter τ_c defines the pulse duration, but does not quantitavely represent it and α is the parameter smoothing the edges of the rectangular aperture in the spectral domain. To approximate the pulses from the experiment, we set $\alpha = 0.05$ and $\tau_c = 0.0128\tau_\mathrm{X}$ and $\tau_c = 0.0064\tau_\mathrm{X}$. These chosen values of τ_c correspond to the same temporal full width at half maximum as the two Gaussian pulses investigated in the main manuscript. In Supplementary Figure 4a, we plot the normalized intensity in dependence of time of the carved pulse of duration $\tau_c = 0.0128\tau_\mathrm{X}$ compared to the Gaussian pulse of duration $0.02\tau_\mathrm{X}$. While the main peak of the carved pulse mimics the Gaussian pulse, the sinc function results in the appearance of sidelobes. These sidelobes result in a small portion of the pulse area being temporally stretched out compared to the perfect Gaussian pulse.

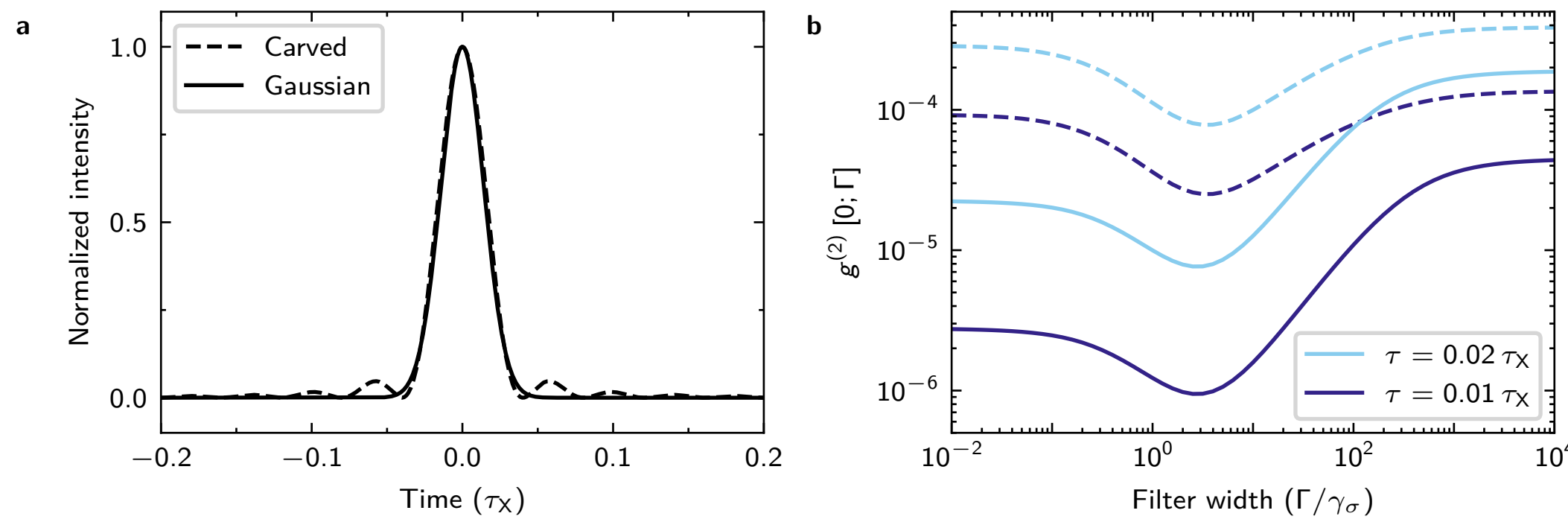

Supplementary Figure 4: Degree of second-order coherence for carved pulses. **a**, Normalized intensity of a Gaussian (solid line) and carved pulse (dashed line). While the main peak of the carved pulse mimics the Gaussian, sidelobes emerge at larger time delays from the pulse center. **b**, Simulated degree of second-order coherence at zero time delay in dependence of the filter width for Gaussian pulses (solid lines) and carved pulses (dashed lines). The pulse duration is chosen to be equal for both pulse shapes (indigo and cyan).

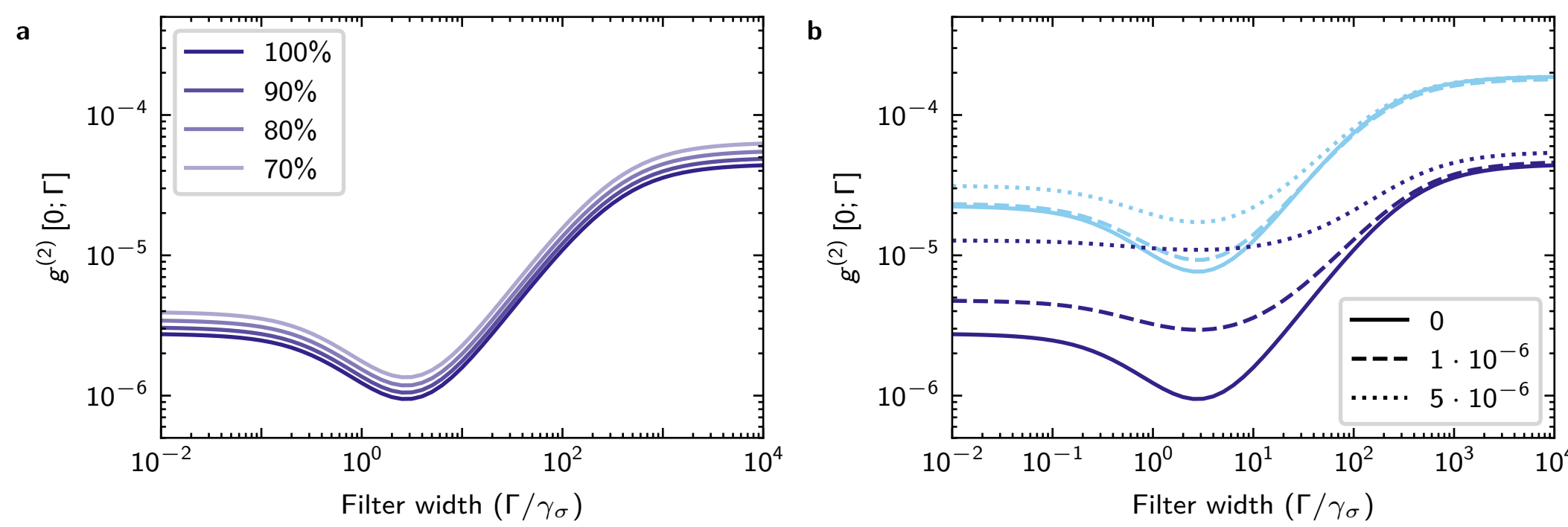

Supplementary Figure 5: Effect of preparation fidelity and shot noise. **a**, Simulated degree of second-order coherence in dependence of the filter width for a $0.01\tau_X$ long driving pulse and preparation fidelities ranging from 100% (higher opacity) to 70% (lower opacity). **b**, Simulated degree of second-order coherence in dependence of the filter width for relative shot noise strengths of 0 (solid lines), $1 \cdot 10^{-6}$ (dashed lines) and $5 \cdot 10^{-6}$ (dotted lines).

How this affects the degree of second-order coherence can be seen in Supplementary Figure 4b, where we plot the $g^{(2)}[0;\Gamma]$ in dependence of the filter width for Gaussian pulses (solid lines) and carved pulses (dashed lines). We observe that for the carved pulses, the absolute $g^{(2)}[0;\Gamma]$ values are higher. In the experimentally accessible region the shape is qualitatively similar, with a plateau for wide filters and a significant improvement in $g^{(2)}[0;\Gamma]$ for decreasing filter widths. This finding confirms that the exact temporal shape of the driving field significantly influences the absolute values of the degree of second-order coherence. Especially the way we derive the pulses in the experiment results in small but observable broad tails in the autocorrelation measurements used to characterize them. Thus, we expect the experimentally measured degree of second-order coherence to be higher than in the theoretical model, as it is also observed in the measurements presented in the manuscript. Note that in experimental measurements of the reappearance of the Rabi rotations in semiconductor quantum dots, the temporal shape of the driving field was also identified to play a role in reaching agreement between theoretical calculations and the experiment [4].

SUPPLEMENTARY NOTE 6: INFLUENCE OF PREPARATION FIDELITY AND UNCORRELATED NOISE SOURCES

Furthermore, we state in the manuscript, that imperfect preparation fidelity also degrades the degree of second-order coherence. This can be caused, e.g., by phonon-induced dephasing during the driving. If the prepared state after the pulse contains a vacuum component, this will scale the effective degree of second order coherence at zero time delay as $g^{(2)}[0;\Gamma]/\eta$, where η is the preparation fidelity [5]. In Supplementary Figure 5, we plot the simulated degree of second-order coherence in dependence of the filter width for different preparation fidelities and a Gaussian driving

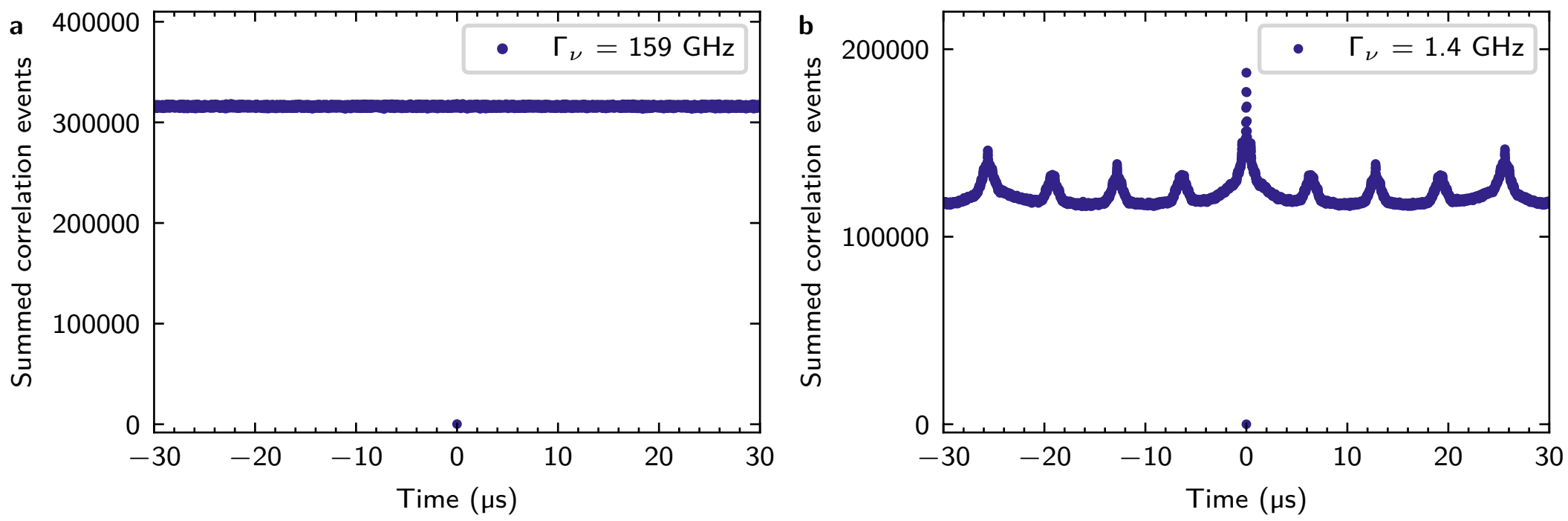

Supplementary Figure 6: Blinking analysis. **a**, Summed counts of the individual peaks of the autocorrelation measurement with the filter width $\Gamma_\nu = 159\,\mathrm{GHz}$ are constant for all side peaks. Only the center peak at zero time delay differs. **b**, Summed counts of the individual peaks for the filter width $\Gamma_\nu = 1.4\,\mathrm{GHz}$ show a periodic blinking on a microsecond timescale.

field of $\tau = 0.01\tau_\mathrm{X}$. We observe that increasing vacuum components degrade the $g^{(2)}[0;\Gamma]$. For the biexciton-exciton cascade in semiconductor quantum dots, a preparation fidelity of 0.91 has been measured [6]. Therefore, we expect our experimentally measured degree of second-order coherence to be also increased by non-unity preparation fidelity.

Lastly, we identified uncorrelated noise sources to potentially further deteriorate the measured $g^{(2)}[0;\Gamma]$. Such noises could for example be detector dark counts or residual laser light, which we discussed already in Supplementary Note 3, or Poissonian shot noise. To discuss its effect, we simulate the degree of second-order coherence and include Poissonian shot noise with the spectral profile of the emission. This results in the simulated degree of second-order coherence for the two studied pulse durations displayed in Supplementary Figure 5b in dependence of the filter width. Here, the solid curves are without any shot noise present as in the manuscript, while the dashed lines include shot noise of relative strength $1 \cdot 10^{-6}$ and the dotted lines of relative strength $5 \cdot 10^{-6}$. We observe that with increasing relative shot noise strength, the $g^{(2)}[0;\Gamma]$ increases. Due to its uncorrelated nature, for large relative strengths it weakens the improvement in $g^{(2)}[0;\Gamma]$, since the increase in shot noise decreases the signal-to-noise ratio. While for decreasing filter widths, the instantaneously emitted photon is still suppressed, for large relative strenths the shot noise starts to dominate over the improvement in $g^{(2)}[0;\Gamma]$. Since we do not observe this in the experimental data, we infer that the impact of shot noise in our experiment is limited. While we identify uncorrelated noise sources such as laser background, detector dark counts and shot noise to not dominate our experimental measurements, they potentially have a deteriorating effect on the measured degree of second-order coherence at zero time delay, increasing the mismatch between the experiment and theory.

SUPPLEMENTARY NOTE 7: BLINKING

As discussed in the manuscript, the studied sample is subject to periodic electric noise which results in blinking for the narrowest filter as displayed in Supplementary Figure 6. We investigate this by summing the counts of all individual peaks of the correlation measurement. For the wider filter of $\Gamma_\nu = 159\,\mathrm{GHz}$ this exhibits a flat line, as seen in Supplementary Figure 6a. This means that we observe no blinking in this measurement configuration. In contrast, for the very narrow etalon filter measurement in the manuscript, we observe a very clear and periodic blinking in the second-order intensity autocorrelation measurement on microsecond timescales as seen in Supplementary Figure 6b. Because of the periodicity and multiple discrete frequencies in the MHz range, we attribute this blinking to periodic electric noise affecting the quantum dot. Together with the quantum confined Stark effect [7], this leads to spectral diffusion on MHz timescales. When the emission is narrowly spectrally filtered, this becomes visible as the observed periodic bunching. The electric noise acting on the quantum dot can be minimized by improved wire shielding, using electric lowpass filters, and lower noise DC sources to bias the diode structure. If the residual electric noise is minimized until the emission linewidth becomes Fourier-limited, this also will inhibit spectral diffusion and result in the restoration of the constant summed correlation events for each individual peak on long timescales. This would remove the decrease of the pulse-wise summed counts of the neighboring peaks for increasing time delay, potentially allowing for the measurement of even lower $g^{(2)}\,[0;\Gamma_\nu]$ values.

[1] S. Bermúdez-Feijóo, E. Zubizarreta Casalengua, K. Müller, and K. D. Jöns, Spectral correlations of dynamical resonance fluorescence, Physical Review Research **7**, 033296 (2025).

[2] P. A. Dalgarno, J. M. Smith, B. D. Gerardot, A. O. Govorov, K. Karrai, P. M. Petroff, and R. J. Warburton, Dark exciton decay dynamics of a semiconductor quantum dot, physica status solidi (a) **202**, 2591 (2005).

[3] A. M. Weiner, J. P. Heritage, and E. M. Kirschner, High-resolution femtosecond pulse shaping, Journal of the Optical Society of America B **5**, 1563 (1988).

[4] L. Hanschke, T. K. Bracht, E. Schöll, D. Bauch, E. Berger, P. Kallert, M. Peter, A. J. Garcia, S. F. Covre Da Silva, S. Manna, A. Rastelli, S. Schumacher, D. E. Reiter, and K. D. Jöns, Experimental Measurement of the Reappearance of Rabi Rotations in Semiconductor Quantum Dots, Physical Review Letters **135**, 263602 (2025).

[5] P. Grünwald, Effective second-order correlation function and single-photon detection, New Journal of Physics **21**, 093003 (2019).

[6] M. B. Rota, F. Basso Basset, D. Tedeschi, and R. Trotta, Entanglement teleportation with photons from quantum dots: Towards a solid-state based quantum network, IEEE Journal of Selected Topics in Quantum Electronics , 1 (2020).

[7] R. J. Warburton, C. Schäflein, D. Haft, F. Bickel, A. Lorke, K. Karrai, J. M. Garcia, W. Schoenfeld, and P. M. Petroff, Optical emission from a charge-tunable quantum ring, Nature **405**, 926 (2000).
