# Supplementary material for: Selective filtering of multi-photon events from a single-photon emitter
Source: Nat Commun. 2026 Aug 14;17:8412. doi: 10.1038/s41467-026-76358-x (PMC13476227; doi:10.1038/s41467-026-76358-x)
Supplement: Supplementary file 2 [file 41467_2026_76358_MOESM2_ESM.pdf]

nature portfolio

Peer Review File

Selective filtering of multi-photon events from a single-photon emitter

Corresponding Author: Mr Friedrich Sbresny

This manuscript has been previously reviewed at another journal. This document only contains information relating to versions considered at Nature Communications.

This file contains all reviewer reports in order by version, followed by all author rebuttals in order by version.

Version 0:

Reviewer comments:

Reviewer #1

(Remarks to the Author)
I have now reviewed the revised manuscript and the authors' response to the referee reports. While the authors have made some effort to address my concerns, I remain unconvinced that the central issue has been resolved. My primary concern is that the work offers limited novelty from a theoretical standpoint. A convincing agreement between theory and experiment could have strengthened the manuscript and provided greater credibility to the claims. However, given the order-of-magnitude discrepancy between the theoretical predictions and the experimental results reported in the paper, I do not find the work sufficiently compelling for publication in Nature Communications.

In their response, the authors provide several possible explanations for the large discrepancy in the data, and some of these explanations may indeed be plausible. Nevertheless, my overall interpretation is that the experimental approach, as implemented, has not produced sufficiently reliable or convincing results. For this reason, I maintain my original assessment that the manuscript is not suitable for publication in a journal aimed at a broad scientific readership, and as such, I recommend its rejection. Perhaps a journal like Scientific Reports would be better suited for publishing the results.

Reviewer #2

(Remarks to the Author)
The authors have now satisfactorily addressed all of my concerns. I believe that the work can be published in its current form in Nature Communications.

Open Access This Peer Review File is licensed under a Creative Commons Attribution 4.0 International License, which permits use, sharing, adaptation, distribution and reproduction in any medium or format, as long as you give appropriate credit to the original author(s) and the source, provide a link to the Creative Commons license, and indicate if changes were made.
In cases where reviewers are anonymous, credit should be given to 'Anonymous Referee' and the source.
The images or other third party material in this Peer Review File are included in the article's Creative Commons license, unless indicated otherwise in a credit line to the material. If material is not included in the article's Creative Commons license and your intended use is not permitted by statutory regulation or exceeds the permitted use, you will need to obtain permission directly from the copyright holder.
To view a copy of this license, visit https://creativecommons.org/licenses/by/4.0/

REVIEWERS' COMMENTS

Reviewer #1 (Remarks to the Author):

I have now reviewed the revised manuscript and the authors' response to the referee reports. While the authors have made some effort to address my concerns, I remain unconvinced that the central issue has been resolved. My primary concern is that the work offers limited novelty from a theoretical standpoint. A convincing agreement between theory and experiment could have strengthened the manuscript and provided greater credibility to the claims. However, given the order-of-magnitude discrepancy between the theoretical predictions and the experimental results reported in the paper, I do not find the work sufficiently compelling for publication in Nature Communications.

In their response, the authors provide several possible explanations for the large discrepancy in the data, and some of these explanations may indeed be plausible. Nevertheless, my overall interpretation is that the experimental approach, as implemented, has not produced sufficiently reliable or convincing results. For this reason, I maintain my original assessment that the manuscript is not suitable for publication in a journal aimed at a broad scientific readership, and as such, I recommend its rejection. Perhaps a journal like Scientific Reports would be better suited for publishing the results.

We thank the referee for reviewing our revised manuscript and their feedback regarding our explanations for the discrepancy between the theoretical calculations and the experimental results.

In our manuscript, we report on a new and important effect for quantum emitters, that is relevant for all quantum systems and many applications. The provided theory is novel and generally applicable to any quantum system. Accordingly, we kept the theory in the manuscript in its most general form. The experiments in the paper demonstrate that the studied effect is relevant, by experimentally observing its signature in the emission of a semiconductor quantum dot.

Experimental imperfections in the form of spectrally complex filter transmission functions, non-ideal Gaussian driving fields, imperfect preparation fidelity and uncorrelated noise sources, reduce the quantitative agreement between the general theoretical model and the experimental results. Importantly, the exact composition of these mechanisms in the experiment is complex and will differ for each individual real-world quantum system and experiment. Nevertheless, as it is important to understand the effect of these imperfections and to serve as a guide for scientists working on many different quantum systems, we now provide a comprehensive discussion of each individual mechanism that we identified to affect the experimentally measured degree of second-order coherence in the Supplementary Information. Additionally, we included a clear statement on the discrepancy in the concluding remarks.

Reviewer #2 (Remarks to the Author):

The authors have now satisfactorily addressed all of my concerns. I believe that the work can be published in its current form in Nature Communications.

We thank the referee for reviewing our revised manuscript and are happy to read that they recommend its publication in Nature Communications in its current form.
